# Comparative Evaluation of Color Stability in Bioactive and Conventional Resin Cements Under Thermal Stress Conditions

**DOI:** 10.3390/biomimetics10070432

**Published:** 2025-07-01

**Authors:** Alaa Turkistani, Hanin E. Yeslam

**Affiliations:** 1 Department of Restorative Dentistry, Faculty of Dentistry, King Abdulaziz University, Jeddah 21589, Saudi Arabia; 2 Advanced Technology Dental Research Laboratory, King Abdulaziz University, Jeddah 21589, Saudi Arabia

**Keywords:** bioactive, resin cements, dental esthetics, color, whiteness

## Abstract

Bioactive resin-based cements (RBCs) were recently introduced, but data on their color stability remain limited. This study analyzed the impact of thermal cycling on the color and whiteness of bioactive RBCs. Specimens (n = 10) were fabricated from Panavia SA Universal (PN), Predicta Bioactive Cement (PR), and ACTIVA BioACTIVE cement (AC). CIE Lab* values were registered at baseline and after 5000, 10,000, and 15,000 thermal cycles (5–55 °C). Changes in color (ΔE_00_) and whiteness index (ΔWI_D_) were calculated and compared. Material type and thermal cycling significantly affected ΔE_00_ and ΔWI_D_ (*p* < 0.001). AC showed the highest ΔE_00_ values at all stages (*p* < 0.001), with a progressive increase over time. PN differed significantly between early and later cycles (*p* < 0.05), while PR remained stable (*p* > 0.05). Analysis of color parameters indicated that AC underwent the most pronounced changes, particularly in Δa and Δb, while PN exhibited the greatest shift in Δb. For ΔWI_D_, PR had significantly lower values than PN (*p* < 0.05) and AC (*p* < 0.001), with no difference between PN and AC (*p* > 0.05), and thermal cycling significantly affected all groups, with PR and AC differing across all stages (*p* < 0.05). Thermal cycling significantly influenced the color stability and whiteness of bioactive RBCs, with AC exhibiting the greatest changes over time, while PR demonstrated superior stability.

## 1. Introduction

Biomimetics is a multidisciplinary field focused on replicating natural tissue components and functions to create innovative materials and systems that utilize nature’s efficient designs for practical applications [1]. In restorative dentistry, biomimetic approaches have revolutionized the restoration of dental defects using materials that emulate natural teeth [2,3]. By adhering to biomimetic principles in adhesive restorative dentistry, advanced composite restorative materials preserve tooth integrity while achieving restorations with optimal esthetics, mechanical performance, and durability [4,5]. A critical aspect of biomimetic restorative dentistry is achieving strong and durable bonds between restorations and tooth structures, mirroring nature’s precision in tissue integration [6]. 

The success of biomimetic restorations depends critically on resin-based cements (RBCs) that replicate dentin’s adaptive bonding mechanisms [7]. These materials offer structural integrity through durable retention and resistance to masticatory forces and marginal seal by preventing microleakage and, subsequently, secondary caries [7,8,9]. RBCs can be classified into one of three categories depending on their bonding mechanism and manipulation technique—adhesive (multistep), self-adhesive (single-step), and universal cements—which may be used in either single- or multi-step procedures [10]. By aligning with biomimetic principles, RBCs help preserve the natural tooth structure while ensuring the restoration’s longevity and functionality.

Various cement-related factors can impact how bonded restorations mimic natural tooth structure, including their esthetics, durability, and mechanical properties [11,12,13]. The light transmission properties of RBCs, which are primarily composed of methacrylates, are influenced by their chemistry [14]. Several RBC components, such as fillers, pigments, opacifiers, and photoinitiators, can influence the color of both the cement and the bonded restoration [9,10,15]. This effect is particularly significant in situations involving thin indirect restorations or in those made from highly translucent materials. In these cases, a thicker layer of cement can further impact the final appearance [9,16]. RBCs are available in a wide range of shades and translucencies, allowing clinicians to select the most suitable option based on the specific patient needs, especially in cases with high esthetic demand [11,16].

Intraoral environment-related factors, such as thermal, mechanical, and chemical challenges, can also affect the properties of dental RBCs [9,10]. To comply with the biomimetic approach in restorative dentistry, RBCs must be able to withstand these challenges [6]. Despite their good bonding and chemo-mechanical properties, they tend to absorb water and undergo hydrolytic changes that eventually affect their mechanical and esthetic stability [5]. This is particularly relevant in restorations with subgingival or equigingival margins that are exposed to crevicular fluid [17]. Ingested foods and beverages with varying temperatures, pigments, and pH levels further challenge the color stability of resin cements [5,8,18]. Additionally, water sorption and hydrolysis can lead to discoloration, microleakage, and eventual debonding of the restoration [19].

Biomimetic advances in RBCs can lead to their further classification into conventional or bioactive types, depending on their bioactive potential [6]. The term “bioactivity” remains a debatable matter in dentistry [20]. Therefore, its use has been regulated in the 2023 FDI World Dental Federation statement along with statements from other organizational reports, including the International Association for Dental Research and the Academy of Dental Materials [21]. The use of the term in the literature generally refers to a biomaterial’s ability to elicit a biological reaction at the interface between the biomaterial and the living tissues [22]. In restorative dentistry, bioactivity generally includes the ability of the restorative material to influence the precipitation of apatite-like crystals when exposed to saliva and/or crevicular fluid, release calcium and phosphate ions to induce remineralization, and/or have antibacterial properties (such as antibacterial nano-fillers) that could prevent the formation of secondary caries lesions at the restorative margin [22,23,24].

Recently, several RBCs with bioactive properties, exemplifying a biomimetic approach, have been introduced into the market for the cementation of esthetic restorations. The components of these cements harness an ion recharge potential which, in turn, helps reduce bacterial microleakage and enhance marginal integrity [25]. ACTIVA BioACTIVE cement (AC; Pulpdent Corporation, Watertown, MA, USA) is essentially a rubberized polymeric bioactive material that combines silica glass particles with a calcium-, phosphate-, and fluoride-enriched polymer matrix [8,23]. This unique blend enhances the cement’s durability, bacterial resistance, microleakage resistance, and bonding to dentin [8,25]. Predicta Bioactive Cement (PR; Parkell, Inc., Edgewood, NY, USA) is yet another recently introduced RBC marketed as a bioactive luting RBC that possesses the ability of ion recharge and induces the deposition of a thick hydroxyapatite layer [26]. PR and similar RBCs containing methacryloyloxydecyl dihydrogen phosphate (10-MDP) are capable of bonding firmly to zirconia-based restorations [27]. In a 2021 study by Al-Saleh et al., AC had better color stability in response to coffee exposure than that of calcium aluminate-based bioactive RBCs with ceramic ionomers [8]. Yet, a study by Mancuso et al. found no difference in the color stability of different types of RBCs in response to water storage [17]. However, bioactive RBCs were not included in that study. In general, research data relevant to the esthetic performance of polymeric bioactive luting cement is limited. To the authors’ knowledge, there are no studies investigating the effect of thermal aging on the color stability and whiteness of different bioactive RBCs.

Given the critical role played by the shade and color of RBCs on the final esthetic quality of bonded restorations and the fact that chemical and bioactive variabilities of different RBCs can potentially affect their color stability, the current study aimed at evaluating the effect of thermal aging on the color and whiteness stability of a conventional dual-cured RBC and two bioactive RBCs. The null hypotheses of the study were that the color stability of bioactive RBCs is not affected by thermal aging and that there are no differences in color stability between the conventional and bioactive RBCs.

## 2. Materials and Methods

A total of 30 disc-shaped specimens (8 × 1 mm) were fabricated from three self-adhesive RBCs (n = 10): a conventional dual-cured RBC (Panavia SA Universal [PN]; Kuraray Noritake Dental, Tokyo, Japan) and two dual-cure bioactive RBCs (AC and PR). A priori power analysis was conducted using G*Power software (G*Power Version 3.1.9.7, Franz Faul, Universität Kiel, Germany) for a repeated-measures ANOVA based on a medium effect size (f = 0.27), an α level of 0.05, and a desired power of 0.80. The analysis indicated a total sample size of 30 specimens, with 10 specimens per material group. Details of the RBCs tested in the study are summarized in Table 1.

A 1 mm thick Teflon rectangular mold with a through circular hole (8 mm in diameter) was placed over a glass slab. The hole was carefully filled with cement, avoiding gross overfilling of the mold. Then, a glass slide was applied on top with slight pressure to prevent the formation of porosities or air bubbles within the cured specimen. The disc-shaped specimens were light-cured with a light-emitting diode (LED) curing unit (E-Morlit, Apoza, New Taipei, Taiwan, ROC) on both the top and bottom surfaces. The curing unit was checked using a spectroradiometer to ensure a consistent irradiance of 1200 mW/cm^2^. After completing the light curing process, the glass slide/slab and Teflon mold were carefully removed, and gross excess at the specimen’s margins was removed with a No. 11 scalpel. All specimens were checked visually under 2.5× magnification and good lighting conditions (illuminant D65) for any obvious voids, air bubbles, cracks, or other defects. Faulty specimens were discarded. Specimens were then left undisturbed and submerged in deionized water at room temperature in a dark container for 24 h to allow for post-curing polymerization completion. The thickness of each disc-shaped specimen was checked using a digital micrometer with an accuracy of ±0.01 mm (Vernier caliper; Hi-Wendy, New Taipei, Taiwan) to ensure a specimen thickness of 1.0 ± 0.1 mm. The bottom surface of each specimen was labeled, and the top surface was left clear for subsequent color measurements.

**Figure 1:**
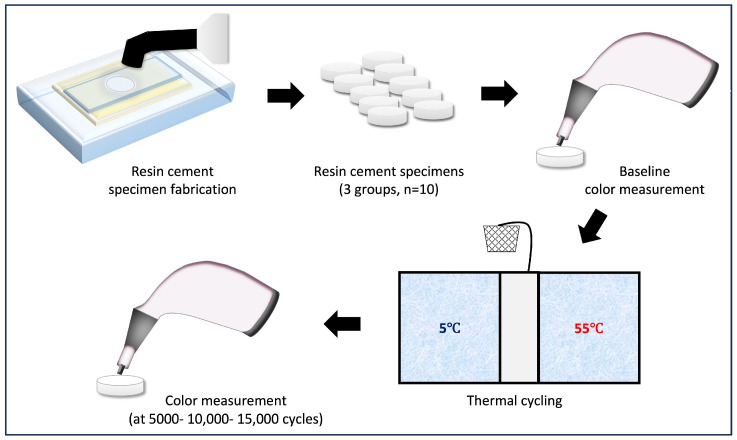
Study design.

A hand-held spectrophotometer (VITA Easyshade Advance, VITA Zahnfabrik, Bad Säckingen, Germany) was used for spectrophotometric analysis [28,29]. Color registration was conducted for all specimens under the same lighting conditions (illuminant D65) by positioning the spectrophotometer tip on the center of the top surface of each specimen. A GC Initial^®^ LiSi block (shade A2), which is a fully crystallized lithium disilicate glass-ceramic computer-aided design and computer-aided manufacturing (CAD/CAM) material, was used as a background under the bottom surface of each specimen for the registration of the color values according to the Commission International de L’Eclairage (CIE) CIELab* and CIEDE2000 color coordinate systems. After performing baseline color measurement, specimens were placed in the thermocycling machine with alternating water baths between 5 °C and 55 °C for a total of 15,000 cycles (Thermocycler THE-1100; SD Mechatronik, Feldkirchen-Westerham, Germany). After each 5000 cycles, specimens were removed from the water bath, rinsed with deionized water, dried with oil-free air spray, and then subjected to spectrophotometric measurements using the same procedure as previously described. The study design is demonstrated in Figure 1.

To calculate the color changes (∆E_00_) after each 5000-cycle interval, the following CIEDE2000 formula was used [30]:

$${∆E}_{00}=\sqrt{{\left(\frac{\Delta L\prime }{K_{L}S_{L}}\right)}^{2}+{\left(\frac{\Delta C\prime }{K_{C}S_{C}}\right)}^{2}+{\left(\frac{\Delta H\prime }{K_{H}S_{H}}\right)}^{2}+R_{T}\;\left(\frac{\Delta C\prime }{K_{C}S_{C}}\right)\left(\frac{\Delta H\prime }{K_{H}S_{H}}\right)}$$

where Δ*L*′ = lightness difference; Δ*C*′ = chroma difference; Δ*H*′ = hue difference; S_L_, S_C_, S_H_= weighting functions for lightness, chroma, and hue; k_L_, k_C_, k_H_ = parametric weighting factors; and R_T_ = a rotation term accounting for interactions between chroma and hue.

To calculate the change in whiteness (∆WI_D_), the following formula was used [31]:

$${∆WI}_{D}=0.511\;{∆L}^{*}-\;2.324\;{∆a}^{*}-\;1.100\;{∆b}^{*}$$

The ΔWI_D_ whiteness change was analyzed according to differences in the CIEL*a*b* lightness (∆L), chroma (∆a), and hue values (∆b) [32]:

∆Lx* = Lx − L baseline; ∆ax* = ax − a baseline; ∆bx* = bx − b baseline

where x is the number of thermal cycles completed; L* is lightness, with 100 indicating white, and zero indicating black; a* is the redness (+ve) and greenness (−ve); and b* is yellowness (+ve) and blueness (−ve).

All data were collected, tabulated, and subjected to statistical analysis. The ΔE_00_ and ΔWI_D_ values were calculated for each material at three measurement stages relative to the baseline: ΔE_00_-1 and ΔWI_D_-1 represent the color difference between the baseline and 5000-cycle interval, ΔE_00_-2 and ΔWI_D_-2 represent the color difference between the baseline and 10,000-cycle interval, and ΔE_00_-3 and ΔWI_D_-3 represent the color difference between the baseline and 15,000-cycle interval.

Statistical analysis of the data was completed using the software for statistical analysis SPSS (IBM SPSS Statistics, v20.0; IBM Corp, Armonk, NY, USA), while Microsoft Office Excel was used for data handling and graphical presentation. Quantitative variables (mean, standard deviation (SD), the range (minimum–maximum), standard error (SE), 95% confidence interval of the mean, and coefficient of variation (CV)) were tabulated (Appendix A). The Shapiro–Wilk test of normality was used to test the normality hypothesis of all quantitative variables to choose the appropriate parametric and non-parametric tests. Most variables were found to be normally distributed, allowing the use of parametric tests (ANOVA and Bonferroni comparisons). A general linear model (GLM) repeated-measure one-way analysis of variance (ANOVA) was applied to statistically analyze each material alone. Then, a mixed-model repeated-measure ANOVA was applied for the material, including the between-subject effect and thermal cycles, to study their interaction. The significance level was set at *p* < 0.05. Two-tailed tests were assumed throughout the analysis for all statistical tests.

## 3. Results

**Figure 2:**
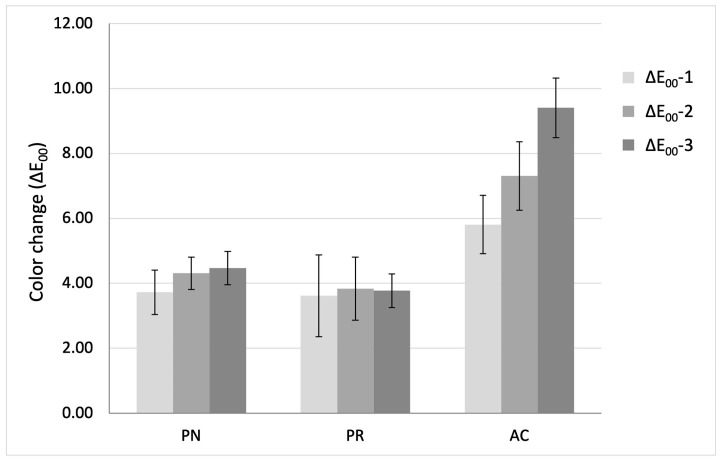
ΔE_00_ values of resin cements at each measurement stage: ΔE_00_-1 (baseline-5000 cycles), ΔE_00_-2 (baseline-10,000 cycles), and ΔE_00_-3 (baseline-15,000 cycles). The black error bars indicate standard deviations.

Repeated-measures mixed-design ANOVA revealed that material type, thermal cycling, and their interaction had a statistically significant effect on ΔE_00_ (*p* < 0.001). Bonferroni pairwise comparisons revealed that AC exhibited significantly higher values than both PN and PR at all measurements (*p* < 0.001). However, the difference between PN and PR was not statistically significant (*p* = 0.69). The different ΔE_00_-1, ΔE_00_-2, and ΔE_00_-3 for all tested RBCs are demonstrated in Figure 2.

Within-group repeated-measure ANOVA for PN and AC indicated a significant difference across measurement stages (*p* = 0.001 and *p* < 0.001, respectively). Bonferroni analysis showed a progressive increase in ΔE_00_ for AC (*p* < 0.001). In PN, a significant difference was observed between ΔE_00_-1 and both ΔE_00_-2 and ΔE_00_-3 (*p* = 0.04 and *p* = 0.003, respectively), whereas the difference between ΔE_00_-2 and ΔE_00_-3 was not significant (*p* = 1.00). For PR, thermal cycling had no significant effect (F = 0.564), as there were no significant differences across the three measurements (*p* > 0.05). Table 2 details the statistically significant differences between the mean ΔE_00_ for each material at the different measurement stages.

Examining the results of the individual color parameters (ΔL, Δa, and Δb) revealed that AC experienced the most pronounced changes in both Δa and Δb, indicating greater chromatic alterations compared to PN and PR. Conversely, PN consistently demonstrated higher Δb values, indicating greater changes along the yellow–blue axis. The differences between the materials became more pronounced with increased thermal cycling. The mean and standard deviation values for individual color parameters are presented in Table 3.

For ΔE_00_-1, the ΔL values were comparable across materials, with PN showing a mean value of 3.1 ± 1.23, slightly lower than PR (3.68 ± 2.38) and AC (3.54 ± 1.27). However, a marked difference was observed in Δa values, where AC demonstrated a higher value (4.83 ± 0.68) compared to PN (0.39 ± 0.27) and PR (0.35 ± 0.33). Similarly, for Δb, PN exhibited the highest value (6.19 ± 0.79), followed by PR (4.11 ± 0.76), while AC showed a negative shift (−2.73 ± 0.59), indicating a change in the blue–yellow axis.

For ΔE_00_-2, ΔL values slightly decreased for all materials compared to the previous stage. PN and PR displayed similar values (2.86 ± 1.01 and 2.61 ± 2.39, respectively), while AC remained slightly higher (3.46 ± 1.32). In the Δa parameter, AC continued to exhibit higher values (6.27 ± 0.89) compared to PN (0.74 ± 0.30) and PR (0.75 ± 0.36). Regarding Δb, PN retained the highest value (7.87 ± 0.77), followed by PR (5.24 ± 1.04), while AC shifted further in the negative direction (-3.40 ± 0.79).

For ΔE_00_-3, the trend in ΔL values persisted, with AC showing the highest mean value (4.04 ± 1.23), while PN and PR exhibited slightly lower values (2.17 ± 1.21 and 1.93 ± 1.27, respectively). AC also maintained a higher Δa value (7.86 ± 0.81) compared to PN (0.40 ± 0.24) and PR (0.87 ± 0.34). For Δb, PN demonstrated the largest positive change (8.85 ± 0.74), followed by PR (5.83 ± 0.56), while AC showed the largest negative shift (-5.03 ± 0.72).

**Figure 3:**
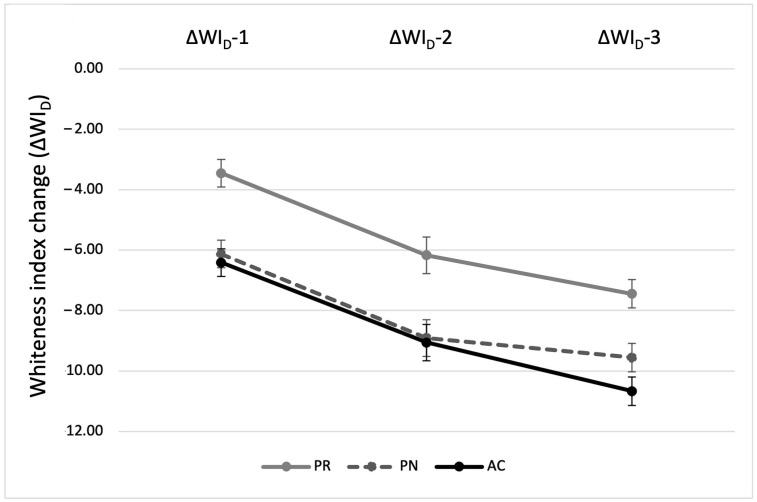
Change in ΔWI_D_ in each material across the measurement stages: ΔWI_D_-1 (baseline–5000 cycles), ΔWI_D_-2 (baseline–10,000 cycles), and ΔWI_D_-3 (baseline–15,000 cycles). The black error bars represent standard deviations.

When ΔWI_D_ values were statistically analyzed, both RBC material type and thermal cycling had a significant effect (*p* < 0.001), while their interaction was not significant (*p* = 0.25). All the RBCs had ΔWI_D_ values above the acceptability threshold (WAT = 2.60 units), which significantly intensified over thermal cycling. At each measurement stage, PR exhibited significantly lower ΔWI_D_ values than PN (*p* = 0.003) and AC (*p* < 0.001), though the difference between PN and AC was not statistically significant (*p* = 1.00). The changes in ΔWI_D_ in the three tested RBCs across the three measurement stages are demonstrated in Figure 3.

Within-group analysis showed a significant effect of thermal cycling on ΔWI_D_ (*p* < 0.001). For PN, ΔWI_D_-1 differed significantly from both ΔWI_D_-2 and ΔWI_D_-3 (*p* < 0.001), with no significant difference between ΔWI_D_-2 and ΔWI_D_-3 (*p* = 0.30). In contrast, both PR and AC exhibited significant differences across all stages, with ΔWI_D_-1 differing significantly from ΔWI_D_-2 and ΔWI_D_-3 (*p* < 0.001), and ΔWI_D_-2 differing from ΔWI_D_-3 (*p* = 0.03 and 0.002, respectively).

## 4. Discussion

This study compared the color and whiteness stability of two bioactive RBCs to conventional RBCs after thermal aging. This combination was chosen to reflect current clinical options and to assess whether the inclusion of bioactive components influences color stability under thermal stress. Comparing these cements allowed us to evaluate whether bioactivity compromises esthetic performance or if certain bioactive formulations can offer both functional and esthetic benefits. Based on the results, thermal aging influenced the color and whiteness of the RBCs tested, and differences were observed between materials at each measurement stage. Accordingly, the two null hypotheses (that color stability is not affected by thermal aging and that no differences in color exist between the RBCs) were rejected.

In this study, shade A2 was consistently selected for the RBCs tested to establish a standardized baseline for color comparison. Additionally, a resin cement thickness of 1 mm was utilized, aligning with International Organization for Standardization (ISO) standards and color testing requirements to ensure reliable results, despite exceeding the typical clinical thickness [33]. To evaluate color stability, both ΔE_00_ and ΔWI_D_ were assessed to provide a comprehensive evaluation reflecting the materials’ esthetic performance clinically. While ΔE_00_ quantifies overall color change, it does not indicate the direction of the shift, such as increased yellowness or reduced brightness, which can be clinically relevant in esthetic restorations, even when ΔE_00_ remains within acceptable limits. Moreover, the recent literature suggests that WI_D_ can be a more sensitive indicator of color degradation in tooth-colored restorative materials, especially under thermal or aging conditions [34].

In addition to ΔE_00_ and ΔWI_D_, ΔL, Δa, and Δb were analyzed individually to identify specific patterns in lightness shifts, chromaticity, and hue alterations to enhance the understanding of how color changed in each material. Clinical relevance was ensured by applying the thresholds of Paravina et al., where ΔE_00_ values between 3.6 and 5.4 were considered moderately unacceptable, and values above 5.4 indicated a clearly or extremely unacceptable color difference [35]. The whiteness thresholds applied in this study were a whiteness perceptibility threshold (WPT) of 0.72 ΔWI_D_ units and a whiteness acceptability threshold (WAT) of 2.60 ΔWI_D_ units, as established by Pérez et al. [34]. All tested RBCs underwent thermal cycling between 5 °C and 55 °C in water baths to simulate intraoral humidity and temperature fluctuations. This process provided an accelerated aging model, with 5000 cycles representing approximately 6 months and 15,000 cycles simulating 18 months of clinical service [36].

In this study, PN was selected as the conventional RBC to which the performance of bioactive RBCs was compared. Thermal cycling to 5000 cycles resulted in a moderately unacceptable color change (ΔE_00_ > 3.72), which intensified at 10,000 cycles but remained stable thereafter. However, WI_D_ showed progressive deterioration over prolonged thermal cycling.

The composition of PN plays a significant role in its color stability and susceptibility to thermal aging. PN is a dual-cure RBC that contains 10-MDP, bisphenol A diglycidyl methacrylate (Bis-GMA), triethyleneglycol dimethacrylate (TEGDMA), and hydroxyethyl methacrylate (HEMA), along with silanated barium glass and colloidal silica fillers. These fillers, ranging in size from 0.02 µm to 20 µm, are treated with silane coupling agents to enhance filler–matrix bonding and mechanical integrity. However, water sorption and hydrolytic degradation remain key factors influencing color stability [37]. The hydrophilic monomer HEMA, along with TEGDMA, contributes to moisture uptake, which can lead to silane hydrolysis and filler debonding, ultimately reducing the cement’s optical stability. Additionally, thermal cycling induces stress and microcracks within the material, further promoting water uptake and solubility, compromising color stability [38]. However, despite these changes, PN’s color in the current study remained more stable compared to the bioactive cement AC, likely due to its stable resin network, filler type and content, and lower water sorption, as smaller filler particles are known to reduce susceptibility to water aging [39]. Additionally, PN’s 10-MDP content might have had a positive effect on its color stability compared to AC, due to its superior cross-linking efficiency during polymerization leading to a denser cured network and a lower water diffusion rate [8,40]. Additionally, 10-MDP was found to reduce reactive oxygen species-induced degradation of the self-adhesive resin matrix [41], which might have also enhanced PN’s color stability. This would suggest the preferable use of PN for the bonding of esthetic restorations in anterior teeth instead of AC. One notable observation in PN’s color shift was the significant increase in Δb values, indicating a yellowing effect over time. This discoloration may be influenced by camphorquinone, the primary light initiator in PN, which is known to become yellowish with aging [42,43]. Additionally, the oxidation of unreacted monomers and residue of unreacted benzoyl peroxide may have contributed to the reduction in WI_D_ over time [44].

Despite the similarity to PN in base components (dimethacrylates, hydroxyethyl methacrylate (HEMA)), PR exhibited a more stable color profile, with less pronounced ΔWI_D_ values over thermal cycling. While the material experienced some color change after 5000 cycles, these changes stabilized and did not deteriorate further, remaining within the range of moderately unacceptable (ΔE_00_ values between 3.6 and 5.4), indicating that the material’s color remained relatively consistent throughout the thermal cycling process. Individual color parameters also showed relatively modest changes over thermal cycling. The observed color and whiteness stability, even under the harsh conditions simulated by 15,000 thermal cycles, indicates the material’s durability in maintaining esthetic outcomes over time.

Bonded dental restorations are routinely exposed to thermal fluctuations and light, including ultraviolet (UV) radiation. The long-term esthetic performance of RBCs under these conditions depends on their specific chemical composition and their tendency to absorb water [45]. Unlike PN, PR contains a novel Poly-2-HEMA monomer, which has reduced hydrophilicity compared to conventional monomers, leading to lower water sorption, improved stability, and consequently, reduced discoloration [46]. In addition, PR’s formulation includes a self-cure initiator system of cumene hydroperoxide and allyl thiourea, which was found to be highly efficient in minimizing yellowing post-curing [47]. Additionally, the presence of 2-Hydroxy-4-methoxybenzophenone, a UV stabilizer [48,49], offers protection against photo-induced discoloration [50], which is critical in maintaining long-term esthetic stability. This stabilizer may also contribute to protecting the resin matrix by reducing the oxidative degradation that can occur during repeated thermal fluctuations in an aqueous environment. 

Among the RBCs tested, AC recorded the highest ΔE_00_-1 value of 5.81, highlighting its susceptibility to thermal aging. As thermal cycling progressed, ΔE_00_ values remained significantly higher than those of the other materials at each measurement stage, indicating a continuous and accelerated esthetic degradation in AC. The observed increase in Δa values suggests a shift toward a reddish hue, which became more pronounced with additional thermal cycles. Unlike other materials, Δb values exhibited a negative trend, suggesting a shift toward a more bluish appearance. However, ΔL values remained relatively stable, emphasizing that the primary color alteration in AC is due to hue and chroma shifts rather than brightness loss. The progressive increase in ΔWI_D_ with additional thermal cycles further underscores the diminished optical stability of AC compared to PN and PR.

These color changes are likely to be due to a combination of material-specific factors, including water sorption, resin hydrolysis, and filler composition. Unlike conventional RBCs, the AC matrix combines diurethane and methacrylates with modified polyacrylic acid. Although this structure improves ion mobility and bioactivity, its high affinity for water leads to increased swelling, plasticization, and structural alterations, making the material more susceptible to discoloration over time. In addition, the specific formulation of AC, including its unique rubberized matrix and high bioactive filler content, could introduce variations in polymer cross-linking density and water permeability, which may have contributed to the progressive color change seen in this study. A previous study demonstrated that ACTIVA BioACTIVE restorative exhibited water sorption values exceeding the ISO limit [51]. Furthermore, a linear relation was found between ΔE_00_ and the amount of water sorption in resin cements, irrespective of polymerization mode [38].

Furthermore, previous studies reported that light-cured RBCs demonstrated superior color stability compared to dual-cure variants, which tended to shift toward red and yellow hues after aging [39]. However, AC exhibited a red shift without a pronounced yellowing effect, likely due to differences in monomer chemistry and polymerization kinetics. Unlike conventional RBCs, AC has three distinct curing mechanisms: light-cure, glass ionomer self-cure, and composite-based self-cure reactions. This hybrid setting mechanism may introduce variations in polymerization efficiency across different regions of the material, potentially affecting its optical uniformity and color stability [39].

Moreover, the bioactive filler content in AC plays a significant role in its esthetic degradation. These fillers are engineered to release and recharge Ca^2+^ and PO_4_^3−^ ions in response to changes in the oral environment [23]. However, repeated thermal cycling alters the solubility and release patterns of these bioactive components. Additionally, the differential thermal expansion coefficients between the resin matrix and bioactive fillers can induce internal stresses, microcrack formation, and filler–matrix debonding, leading to increased porosity and enhanced water sorption [18]. This process not only weakens the polymer matrix network but also exposes new filler surfaces, affecting light scattering and refraction [18,52]. Additionally, scanning electron microscopy (SEM) analysis revealed that ACTIVA BioACTIVE restorative developed a rougher and more microporous surface after thermal cycling [53]. These changes suggest that thermal aging can influence both the ion release capabilities and surface characteristics, exacerbating color instability.

The study results highlighted the differences in color stability of RBCs under thermal stresses, revealing that AC experienced significant color degradation, which potentially can affect the esthetic outcomes of restorations in clinical practice. In contrast, PR showed superior color stability, making it the preferred bioactive choice for high-esthetic cases. Clinicians should carefully consider RBC selection when bonding esthetic restorations to achieve proper functional and aesthetic restoration success, ultimately improving patient satisfaction.

These findings have important clinical implications, particularly in anterior restorations where esthetics are paramount. Discoloration of the underlying resin cement can become visible through translucent ceramic materials over time, especially in cases with minimal thickness. This may lead to compromised esthetic outcomes and potential patient dissatisfaction. The superior stability observed in PR suggests that it may be a more reliable option in such scenarios, whereas materials showing greater discoloration, such as AC, may be better suited for less esthetically critical areas. As such, cement selection should not only be guided by mechanical or bioactive properties but also by long-term esthetic performance. To help mitigate discoloration, clinicians may consider applying surface sealants, minimizing cement thickness in esthetic zones, and optimizing curing protocols to reduce residual monomer content that can contribute to staining over time.

However, several limitations of the present study should be acknowledged. Firstly, only one cement shade (A2) was evaluated. Future studies incorporating a broader range of shades would offer a more comprehensive understanding of color stability across different clinical scenarios. Secondly, color measurements in this study were obtained using instrumental methods (spectrophotometry) without a corresponding visual assessment. Incorporating visual assessments in future studies would help bridge the gap between objective color changes and their esthetic relevance to patients and practitioners.

Additionally, direct morphological analysis (e.g., scanning electron microscopy) could provide further insight into the mechanisms of discoloration, particularly in relation to surface roughness, matrix degradation, or filler leaching. The inclusion of such analyses in future research would deepen our understanding of the structural factors contributing to color instability.

Moreover, the discoloration observed under thermal cycling may not fully represent the complexities of intraoral aging. Variables such as pH fluctuations, mechanical loading, enzymatic activity, and staining from common dietary sources (e.g., coffee, tea) may further compromise the color stability of bioactive cements. Thus, long-term in vivo studies or in vitro studies that simulate comprehensive aging conditions are necessary to enhance the clinical relevance of the findings.

Finally, investigating the interaction between the cement and different types of restorative materials in bonded restorations—rather than isolated cement specimens—would provide a more clinically relevant perspective, as this interface likely influences the overall esthetic appearance.

## 5. Conclusions

This study demonstrated that thermal cycling significantly affects the color stability and whiteness of RBCs, with notable differences among materials. Although both AC and PR are bioactive cements, their performance varied considerably. AC exhibited the greatest change in color and whiteness over time, showing lower color stability than both the PN and the other bioactive cement (PR). In contrast, PR outperformed PN, demonstrating superior color stability across all measurement stages. PN showed moderate color shifts, particularly along the yellow–blue axis.

These findings indicate that color stability in bioactive cements is material dependent. PR’s stability suggests that bioactive properties can be achieved without compromising long-term esthetic performance. Clinically, these results reinforce the importance of selecting resin cements based on both functional and esthetic considerations, particularly in restorations requiring long-term color stability.

## Figures and Tables

**Table 1:** Overview of resin-based cements investigated in this study.

Resin-Based Cement (Shade)	Abbreviation	Manufacturer (Lot #)	Composition	Application Instructions
Panavia SA Universal (A2)	PN	Kuraray Noritake Dental, Tokyo, Japan (#140200)	Paste A: MDP, Bis-GMA, TEGDMA, hydrophobic aromatic dimethacrylate, HEMA, silanated barium glass filler, silanated colloidal silica, dl-camphorquinone, peroxide, catalysts, pigments Paste B: hydrophobic aromatic dimethacrylate, silane coupling agent, silanated barium glass filler, aluminum oxide filler, surface-treated sodium fluoride (less than 1%), dl-camphorquinone, accelerators, pigments	Dispense equal amounts of paste A and B and mix for 10 s. Apply and light cure for 10 s
ACTIVA BioACTIVE cement (A2)	AC	Pulpdent, Watertown, MA, USA (#221118)	Diurethane and other methacrylates with modified polyacrylic acid, silica, sodium fluoride	Place cement and light cure for 20 s
Predicta Bioactive Cement (A2)	PR	Parkell, Edgewood, NY, USA (#23017)	Base component: glass oxide, Bis-GMA, UDMA, HEMA, TMPTMA, BTHQ, calcium fluoride, photoinitiators Catalyst component: 10-MDP, HEMA, UDMA, TMPTMA, cumene hydroperoxide, photoinitiators	Dispense and light cure for 30 s

Abbreviations. Bis-GMA: bisphenol A diglycidyl methacrylate; BTHQ: 2,6-Di-tert-butyl-p-cresol; HEMA: hydroxyethyl methacrylate; MDP: 10-methacryloyloxydecyl dihydrogen phosphate; TEGDMA: triethyleneglycol dimethacrylate; TMPTMA: trimethylolpropane trimethacrylate; UDMA: urethane dimethacrylate.

**Table 2:** Mean and standard deviation of ΔE_00_ for each material at each of the three stages: ΔE_00_-1 (baseline–5000 cycles), ΔE_00_-2 (baseline–10,000 cycles) and ΔE_00_-3 (baseline–15,000 cycles). Values with different upper superscripts within the same horizontal row and lowercase superscripts within the same vertical column are significantly different (*p* < 0.05).

Measurement	(Mean ± SD)		
	PN	PR	AC
ΔE_00_-1 (baseline–5000 cycles)	3.72 ± 0.69 ^Bb^	3.61 ± 1.26 ^Ba^	5.81 ± 0.90 ^Ac^
ΔE_00_-2 (baseline–10,000 cycles)	4.31 ± 0.50 ^Ba^	3.83 ± 0.97 ^Ba^	7.31 ± 1.05 ^Ab^
ΔE_00_-3 (baseline–15,000 cycles)	4.47 ± 0.51 ^Ba^	3.77 ± 0.52 ^Ba^	9.41 ± 0.92 ^Aa^

**Table 3:** Mean values and standard deviations of ΔWI_D_ and color parameters (ΔL*, Δa*, Δb*) across three stages (baseline–5000 cycles; baseline–10,000 cycles; baseline–15,000 cycles). Statistical differences were calculated only for ΔWI_D_. Values with different uppercase superscripts within rows and lowercase superscripts within columns are significantly different (*p* < 0.05).

Measurement		Mean ± SD		
		PN	PR	AC
ΔWI_D_-1 (baseline–5000 cycles)		6.13 ± 0.36 ^Ab^	3.45 ± 0.55 ^Bc^	6.41 ± 0.44 ^Ac^
Color parameters	ΔL	3.1 ± 1.23	3.68 ± 2.38	3.54 ± 1.27
	Δa	0.39 ± 0.27	0.35 ± 0.33	4.83 ± 0.68
	Δb	6.19 ±0.79	4.11 ± 0.76	−2.73 ± 0.59
ΔWI_D_-2 (baseline–10,000 cycles)		8.92 ± 0.39 ^Aa^	6.17 ± 0.74 ^Bb^	9.06 ± 0.63 ^Ab^
Color parameters	ΔL	2.86 ± 1.01	2.61 ± 2.39	3.46 ± 1.32
	Δa	0.74 ± 0.30	0.75 ± 0.36	6.27 ± 0.89
	Δb	7.87 ± 0.77	5.24 ± 1.04	−3.40 ± 0.79
ΔWI_D_-3 (baseline–15,000 cycles)		9.56 ± 0.21 ^Aa^	7.45 ± 0.48 ^Ba^	10.67 ± 0.62 ^Aa^
Color parameters	ΔL	2.17 ± 1.21	1.93 ± 1.27	4.04 ± 1.23
	Δa	0.40 ± 0.24	0.87 ± 0.34	7.86 ± 0.81
	Δb	8.85 ± 0.74	5.83 ± 0.56	−5.03 ± 0.72

## Data Availability

The raw data supporting the conclusions of this article will be made available by the authors on request.

## Abbreviations

The following abbreviations are used in this manuscript:

AC	ACTIVA BioACTIVE cement
ANOVA	One-way analysis of variance
Bis-GMA	Bisphenol A diglycidyl methacrylate
BPA	Bisphenol A
CIE	Commission International de L’Eclairage
GLM	General linear model
HEMA	Hydroxyethyl methacrylate
ISO	International Organization for Standardization
LED	Light-emitting diode
MDP	Methacryloyloxydecyl dihydrogen phosphate
OMB	2-Hydroxy-4-methoxybenzophenone
PR	Predicta Bioactive Cement
RBCs	Resin-based cements
SEM	Scanning electron microscopy
TEGDMA	Triethyleneglycol dimethacrylate
UDMA	Urethane dimethacrylate
UV	Ultraviolet
WAT	Whiteness acceptability threshold
WPT	Whiteness perceptibility threshold

## Appendix A

**Table A1:** Descriptive statistics for the change in color (ΔE_00_) and change in whiteness (ΔWI_D_) for each tested material after each 5000-thermal cycle interval.

		N	Mean	SD	SE	95% Confidence Interval for Mean		Minimum	Maximum	Coefficient of Variation (CV)
						Lower Bound	Upper Bound			
ΔE_00_-1 (baseline–5000 cycles)	PN	10	3.72	0.69	0.22	3.23	4.21	2.90	4.83	18.43%
	PR	10	3.61	1.26	0.40	2.71	4.51	2.06	5.73	34.78%
	AC	10	5.81	0.90	0.28	5.16	6.45	3.63	6.83	15.50%
ΔE_00_-2 (baseline–10,000 cycles)	PN	10	4.31	0.50	0.16	3.95	4.67	3.35	4.97	11.58%
	PR	10	3.83	0.97	0.31	3.14	4.53	2.63	5.50	25.32%
	AC	10	7.31	1.05	0.33	6.55	8.06	5.22	8.70	14.43%
ΔE_00_-3 (baseline–15,000 cycles)	PN	10	4.47	0.51	0.16	4.10	4.84	3.80	5.35	11.52%
	PR	10	3.77	0.52	0.16	3.40	4.14	3.07	4.36	13.78%
	AC	10	9.41	0.92	0.29	8.75	10.06	7.69	10.74	9.75%
ΔWI_D_-1 (baseline–5000 cycles)	PN	10	−6.13	1.13	0.36	−6.94	−5.32	−7.91	−4.11	18.39%
	PR	10	−3.45	1.75	0.55	−4.71	−2.20	−7.48	−1.43	50.66%
	AC	10	−6.41	1.40	0.44	−7.41	−5.41	−8.06	−3.93	21.83%
ΔWI_D_-2 (baseline–10,000 cycles)	PN	10	−8.92	1.24	0.39	−9.80	−8.03	−11.70	−7.58	13.87%
	PR	10	−6.17	2.33	0.74	−7.84	−4.50	−9.88	−2.32	37.81%
	AC	10	−9.06	1.99	0.63	−10.49	−7.64	−12.55	−5.59	21.94%
ΔWI_D_-3 (baseline–15,000 cycles)	PN	10	−9.56	0.67	0.21	−10.04	−9.08	−10.23	−8.21	7.03%
	PR	10	−7.45	1.52	0.48	−8.54	−6.36	−10.05	−4.14	20.47%
	AC	10	−10.67	1.95	0.62	−12.07	−9.27	−13.93	−7.34	18.30%

Abbreviations: CV: coefficient of variation; SD: standard deviation; SE: standard error.
